# Supplementary material for: The interactome of intact mitochondria by cross-linking mass spectrometry provides evidence for coexisting respiratory supercomplexes
Source: Mol Cell Proteomics. 2017 Dec 8;17(2):216–32. doi: 10.1074/mcp.RA117.000470 (PMC5795388; doi:10.1074/mcp.RA117.000470)
Supplement: Supplemental Data [file supp_RA117.000470_133922_0_supp_23978_fzffwf.zip › spectra_annotation/mito_spectra_annotation/89-1-83-1-10-1.pdf]

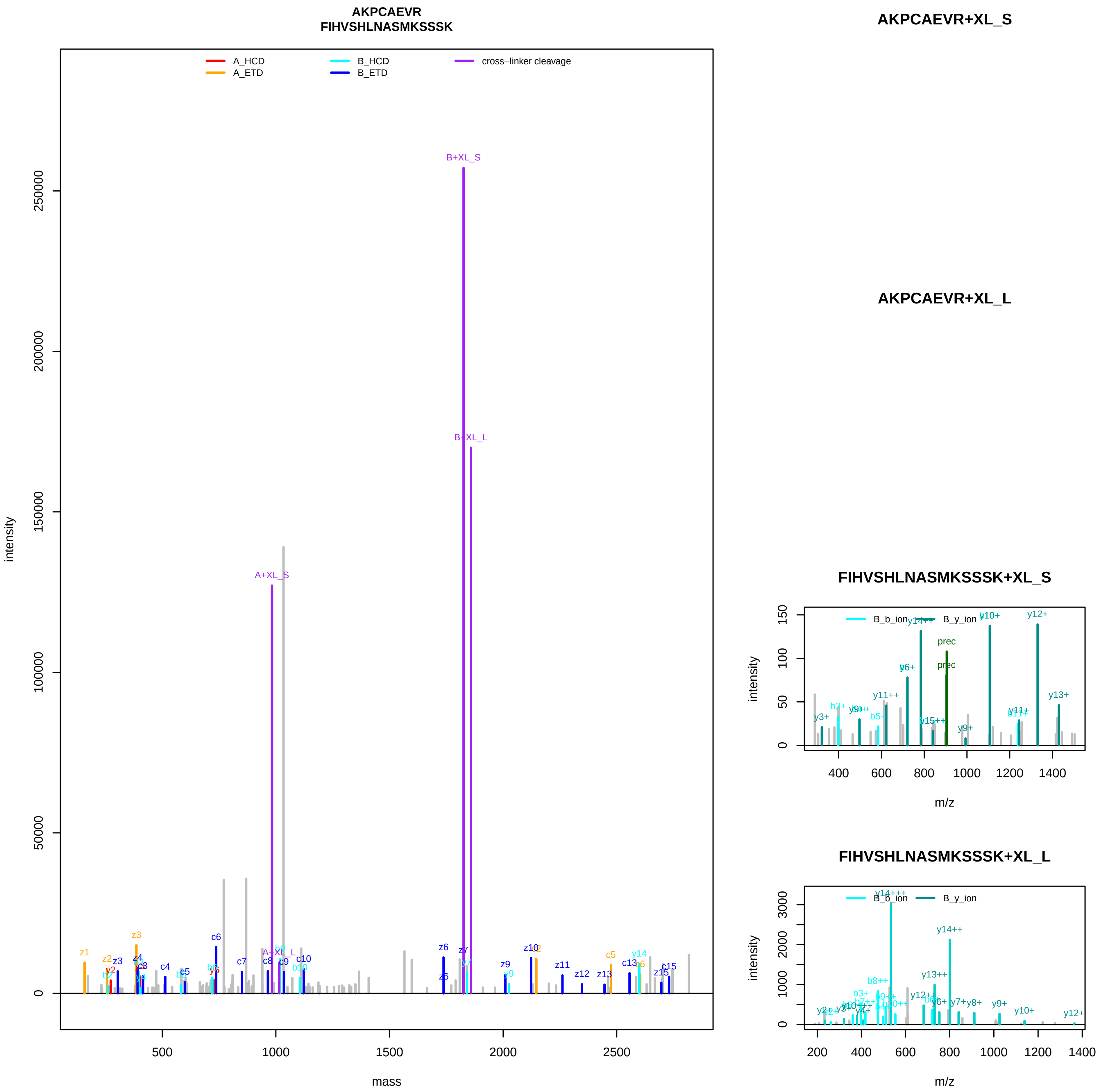

AKPCAEVR
FIHVSHLNASMKSSSK
A_HCD
A_ETD
B_HCD
B_ETD
cross−linker cleavage
B+XL_S
B+XL_L
A+XL_S
A+XL_L
intensity
mass
0
50000
100000
150000
200000
250000
500
1000
1500
2000
2500
z1
z2
z3
y2
z4
c3
c4
c5
c6
y5
c7
c8
c9
c10
b10
z6
z7
y7
z9
y9
z10
z11
z12
z13
c5
c13
y14
c15
z15
AKPCAEVR+XL_S
AKPCAEVR+XL_L
FIHVSHLNASMKSSSK+XL_S
B_b_ion
B_y_ion
y10+
y12+
y14++
prec
y6+
y11++
b3+
y3+
b5+
y15++
y9+
y11+
y13+
intensity
m/z
0
50
100
150
400
600
800
1000
1200
1400
FIHVSHLNASMKSSSK+XL_L
B_b_ion
B_y_ion
y14+++
y14++
y13++
b8++
b3+
y12++
y6+
y7+
y8+
y9+
y10+
y12+
intensity
m/z
0
1000
2000
3000
200
400
600
800
1000
1200
1400
